# Peripheral ulcerative keratitis associated with tralokinumab therapy: a case report and review of IL-13 inhibitor-associated ocular surface disease

**DOI:** 10.1186/s12348-024-00394-1

**Published:** 2024-04-16

**Authors:** Nenita Maganti, Samuel A. Whittier, Evan J. Warner

**Affiliations:** The Department of Ophthalmology and Visual Sciences, University of Wisconsin-Madison, 2870 University Avenue, Suite 206, Madison, WI 53705 USA

## Abstract

**Background:**

Dupilumab and tralokinumab are interleukin-binding monoclonal antibodies used to treat systemic atopic disease. Use of these medications in management of atopic dermatitis (AD) is known to cause conjunctivitis. Dupilumab therapy has also been associated with more severe ocular surface disease, which has not previously been described in association with tralokinumab. This report describes a case of tralokinumab-associated conjunctivitis and peripheral ulcerative keratitis and reviews the spectrum and proposed mechanisms of ocular surface disease triggered by these medications.

**Case presentation:**

A 61-year-old male with no rheumatologic or ocular history presented with bilateral papillary conjunctivitis and right eye peripheral ulcerative keratitis (PUK). PUK was arrested using oral corticosteroids and doxycycline, and long-term control of papillary conjunctivitis was achieved using topical tacrolimus ointment, allowing continuation of effective AD management with tralokinumab.

**Conclusion:**

This case report documents ulcerative keratitis occurring in association with tralokinumab therapy for AD, which has previously been described with dupilumab but not tralokinumab. This report demonstrates the need for vigilant ocular surface monitoring for patients on tralokinumab and illustrates successful management and long-term control of adverse ocular events associated with this medication.

## Introduction

Dupilumab, an interleukin (IL)-4 and IL-13 binding monoclonal antibody (Ab), and tralokinumab-ldrm, an IL-13 binding monoclonal Ab, are human monoclonal IgG4 biologics that have been successful in the treatment of moderate-to-severe atopic dermatitis [1]. Clinical trials of both medications demonstrated increased incidence of conjunctivitis compared with placebo, with dupilumab (28% of treatment group vs. 11% in placebo controls) [2] having much higher incidence of conjunctivitis than tralokinumab (5% of treatment group vs. 2% of controls) [3]. Case series since FDA approval have revealed a substantially higher real-world frequency of dupilumab-associated conjunctivitis and other ocular surface disease [1, 4–8], without such reporting for tralokinumab. While most conjunctivitis identified in association with these medications is mild and responds well to topical therapy, some may be severe or refractory and require cessation of biologic therapy [7]. The ocular surface disease spectrum reported to be associated with dupilumab also includes conjunctival cicatrization, periocular skin changes, punctal stenosis, madarosis, ulcerative keratitis, and corneal perforation [2, 4, 8, 9]. To date, reports of severe ocular surface disease beyond conjunctivitis have not been published in association with tralokinumab therapy. Here, we present a case of severe conjunctivitis and peripheral ulcerative keratitis (PUK) associated with tralokinumab therapy. While dupilumab has been associated with PUK [9] and ulcerative keratitis leading to perforation [2] previously, this is the first known report of ulcerative keratitis associated with tralokinumab.

## Clinical case

A 61-year-old male with no history of rheumatologic or prior ophthalmic disease presented to ophthalmology clinic with acute concerns of profuse tearing, pain, and blurred vision in the right eye. Pain was described as sharp and burning in character, with severity 7/10. General medical history was significant only for atopic dermatitis and hypertension. He had previously been treated for atopic dermatitis with dupilumab (*Dupixent*; Sanofi) injections for nine months, which had been stopped more than two years prior due to lack of efficacy. 18 weeks prior to presentation in eye clinic, he had started therapy with tralokinumab (*Adbry*; LEO Pharma), with an initiation dose of 600 mg subcutaneous injection, followed by maintenance dosing of 300 mg subcutaneous injection every two weeks. The patient reported significant improvement in atopic dermatitis symptoms since starting tralokinumab, but described symptoms of bilateral eye redness, irritation, tearing, and photosensitivity that had been worsening since onset six weeks after starting tralokinumab injections. His ocular symptoms had not improved with the frequent use of non-preserved artificial tears.

Initial examination revealed best-corrected visual acuity (BCVA) of 20/50 in the right eye and 20/40 in the left eye. Intraocular pressure, pupil reactions, confrontation visual fields, and extraocular motility were normal bilaterally. Eyelids and periocular skin showed no active dermatitis, with mild eyelid skin thickening/hypertrophy. Slit lamp examination revealed 2–3 + conjunctival injection and fine papillae on the superior and inferior tarsal conjunctiva bilaterally, with mild punctate epithelial erosions of both corneas. The right cornea had a perilimbal, crescentic infiltrate measuring 1.2 mm wide and spanning from 4 o’clock to 7 o’clock, with an overlying epithelial defect and 20–25% stromal thinning. No anterior chamber reaction was present.

A diagnosis of bilateral papillary conjunctivitis and right eye peripheral ulcerative keratitis (PUK) was made. Cultures of the corneal ulceration were negative. Treatment of active PUK was initiated with oral prednisone burst at 40 mg daily to arrest the immunologic corneal melt, oral doxycycline 100 mg twice daily to suppress matrix metalloproteinases involved in stromal collagenolysis, oral ascorbic acid 500 mg twice daily to stabilize and allow re-building of stromal collagen, and bacterial infection prophylaxis with topical moxifloxacin 0.5% four times daily. A blood draw was done for systemic rheumatologic evaluation prior to starting oral steroid. A comprehensive metabolic panel was entirely within normal limits, and screening for rheumatoid factor, anti-cyclic citrullinated peptide, anti-nuclear antibody, anti-neutrophil cytoplasmic antibody, and angiotensin converting enzyme were all negative. A complete review of systems with focus on rheumatologic disease symptoms was unremarkable.

**Fig. 1:**
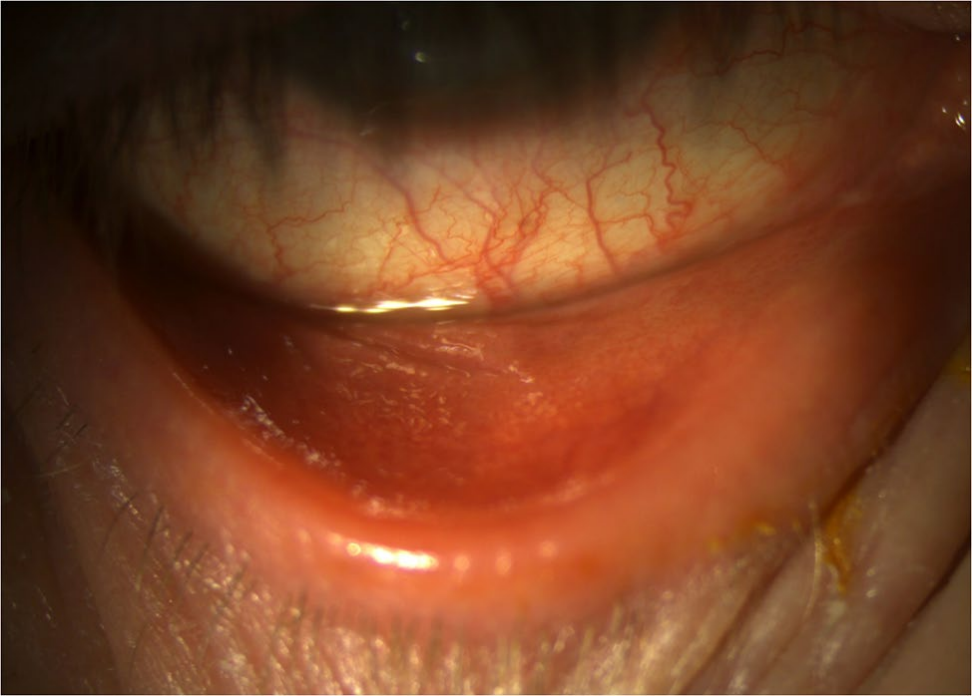
Slit lamp exam reveals diffuse bulbar conjunctival injection and fine papillary conjunctivitis of the palpebral conjunctiva, which had already improved significantly from initial presentation

**Fig. 2:**
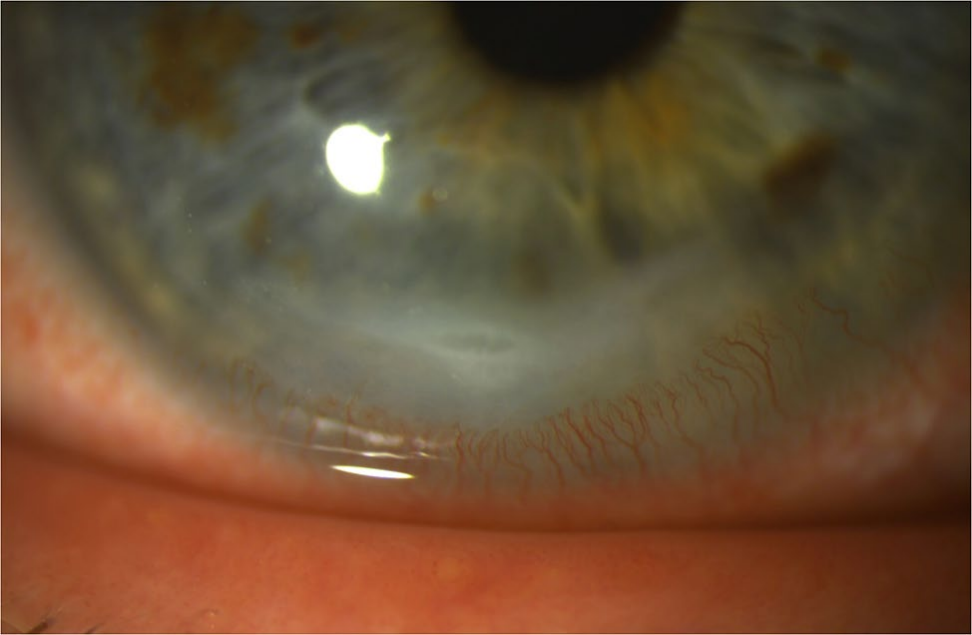
Though improving at the time of photography after three days of oral prednisone therapy, a crescentic corneal infiltrate remains present near the inferior limbus. Significant leukocytic stromal inflammation remains, with reactive corneal neovascularization, a breakdown of corneal epithelium overlying the infiltrate, and loss of 20% stromal thickness

Due to lack of available photography equipment, no imaging was performed at the initial evaluation. At a follow-up examination three days later, slit lamp photos were obtained, documenting moderate and improving papillary conjunctivitis (Fig. 1) and a resolving inferior crescentic corneal infiltrate and ulcerative keratitis (Fig. 2). The corneal epithelial defect had almost fully healed. BCVA was 20/40 in the right eye. Topical moxifloxacin was decreased to twice daily and oral prednisone was maintained at 40 mg per day. Additionally, topical tacrolimus 0.03% was prescribed twice daily to control tralokinumab-associated ocular surface disease and papillary conjunctivitis. At one week from initial presentation, follow-up exam revealed a nearly-resolved inferior crescentic PUK infiltrate with the overlying epithelium fully intact, along with diminishing papillary conjunctivitis and punctate corneal erosions. BCVA in the right eye had improved to 20/25. Oral prednisone was stopped, completing a seven-day total burst. A taper of topical prednisolone 1% was used in the right eye over four weeks, starting at four times daily, to ensure resolution of all corneal inflammation. Oral doxycycline and topical moxifloxacin were stopped since all epithelium was intact. At follow-up one month later, BCVA had returned to 20/20 in each eye, with no active corneal inflammation. Bilateral papillary conjunctivitis and resulting corneal epithelial erosions were resolved. In discussion with the patient and his dermatologist, continued use of tralokinumab was planned given the high efficacy in treating his atopic dermatitis. Options for maintenance control of tralokinumab-associated ocular surface disease were discussed, including topical cyclosporine and lifitigrast, but the patient elected to continue topical tacrolimus 0.03% daily due to good tolerance and efficacy. At final follow-up 4 months later, he maintained good control of atopic dermatitis with ongoing tralokinumab injections and had no recurrence of papillary conjunctivitis or other ocular surface symptom concerns.

## Discussion

Atopic dermatitis (AD) is a common skin condition reported to impact up to one-fifth of individuals during their lifetime, with variable prevalence and severity depending on climate and environment [10]. In developed countries, AD is diagnosed in up to 20% of children and 1–3% of adults [11]. While corticosteroids are the mainstays of AD management, they are only effective in around 20% of patients with moderate-to-severe disease, with treatment options limited by adverse effect such as skin atrophy, pigmentation changes, and hypothalamus-pituitary-adrenal axis suppression [12]. Immunomodulatory therapies have recently emerged as effective options in the treatment of moderate-to-severe AD, with conjunctivitis and other ocular surface disease being identified as the most common adverse events related to such treatment.

Peripheral ulcerative keratitis (PUK) is an inflammatory condition of the cornea that typically presents with perilimbal, crescentic inflammation of the corneal stroma. This inflammation can lead to breakdown of the overlying epithelium and progressive, collagenolytic thinning of the corneal stroma [13]. The peripheral cornea, unlike the avascular central cornea, has a robust vascular supply, allowing for deposition of cellular and molecular inflammatory mediators. Although the exact mechanism of PUK is not fully understood and involves a complex interplay of many factors, both cell-mediated and antibody-mediated immune activation have been implicated in pathogenesis, with inflammatory cells from limbal vasculature triggering complement activation and matrix-metalloproteinase-driven destruction of the corneal stroma [14–16]. PUK may have local triggers including infectious, postsurgical, and traumatic etiologies or be an ocular manifestation of systemic autoimmune disorders, including rheumatoid arthritis, ANCA vasculitides, collagen vascular disease, and inflammatory bowel disease. While autoimmune diseases are associated with more than half of PUK cases, with rheumatoid arthritis being the most common [13], there are increasing reports of ulcerative keratitis occurring in association with immune-modulatory biologics that alter cytokine levels and inflammatory pathways [17]. Our patient’s history of longstanding atopic dermatitis is itself a risk factor for PUK [18], as are other dermatologic conditions such as rosacea, mucous membrane pemphigoid, Stevens-Johnson syndrome, and psoriasis [16, 19]. However, this 61-year-old patient had suffered from atopic dermatitis all his life, with no prior history of any ocular manifestations of atopic disease before starting tralokinumab therapy. Given prior reports of ulcerative keratitis occurring in association with the IL-4 and IL-13 inhibitor dupilumab [2, 9], we hypothesize that the conjunctivitis and ocular surface inflammation known to be triggered by the IL-13 inhibitor tralokinumab may have been a contributing factor in this episode of PUK.

Dupilumab, an interleukin (IL)-4 and IL-13 binding monoclonal antibody (Ab), and tralokinumab-ldrm, an IL-13 binding monoclonal Ab, are human monoclonal IgG4 biologics that have demonstrated efficacy in the treatment of moderate-to-severe atopic dermatitis [1]. Although keratitis and conjunctivitis are more common in patients with atopic disease in general, treatment of atopic dermatitis with these monoclonal antibodies has been noted to worsen these ocular conditions or to trigger them in patients with no prior ophthalmic manifestations of atopic disease [20, 21]. 

A phase 2b study (NCT02347176) demonstrated development of conjunctivitis in 5.9% of patients treated with tralokinumab [22]. ECZTRA 1 and ECZTRA 2 were randomized, double-masked, placebo-controlled clinical trials where conjunctivitis was found to be the most common adverse ocular event seen with tralokinumab treatment. This was more common in the treatment group versus the placebo group, and only subjects receiving tralokinumab were diagnosed with keratoconjunctivitis affecting the corneal epithelium over the course of the trials [23]. In the double-masked, placebo-controlled phase 3 trial (ECZTRA 3), conjunctivitis was noted more in the tralokinumab treatment group (13.1%) than the placebo group (5.6%), though no patients required cessation of the medication [24]. 

Across numerous industry-sponsored clinical trials, conjunctivitis rates associated with dupilumab and tralokinumab range from 3 to 31% and 2–13% respectively.^1^ Case series publications since the clinical launch of these medications have shown a higher proportion of conjunctivitis with dupilumab use, ranging from 43–69%,[4–6,29] with these higher rates of ocular surface disease not being mirrored in the literature regarding tralokinumab. More severe ocular surface disease associated with dupilumab has also been reported, including conjunctival cicatrization, periocular skin changes, punctal stenosis, madarosis, peripheral ulcerative keratitis, and even corneal perforation.[2,4], 8–9, 25−30] Per a review of PubMed, Google Scholar, and Cochrane Database performed on December 18, 2023, severe ocular surface disease beyond conjunctivitis has not been reported in association with tralokinumab therapy.

Although the pathogenic mechanisms of dupilumab and tralokinumab-associated ocular surface disease is not fully elucidated, it had been shown that inhibition of IL-13 prevents conjunctival goblet cells from being activated, inducing goblet cell hypoplasia and reduced mucin secretion [31, 32]. This, in turn, decreases tear film stability and may lead to conjunctivitis via increased surface friction and disruption of the mucosal-epithelial barrier. Barnett et al. [31] has demonstrated mucin deficiency in patients using dupilumab, while Bakker et al. [32] has documented a paucity of conjunctival goblet cells along with increased T-cell and eosinophilic infiltrates in conjunctival biopsies of AD patients diagnosed with dupilumab-associated conjunctivitis. This combination of mucosal-epithelial breakdown and T-cell infiltration provides an environment primed to cascade into keratitis and potentially corneal stromal ulceration.

While there are no current standards for the management of dupilumab and tralokinumab-associated ocular surface disease, many such cases of conjunctivitis are easily managed with topical medications, and cessation of monoclonal antibody therapy is rarely necessary [1]. Counseling and anticipation of potential ocular side effects is important, as is close communication between prescribing dermatologists or allergists and ophthalmic providers. Mild conjunctivitis can be managed using aggressive lubrication and topical antihistamine/mast cell stabilizer drops. In moderate-to-severe conjunctivitis, adding anti-inflammatory therapies such as corticosteroids (prednisolone 1%, fluoromethalone 0.1%), calcineurin inhibitors (cyclosporine 0.05-2%, tacrolimus 0.03–0.1%), or lifitigrast can be highly effective to achieve and maintain inflammatory control [33–36]. More severe forms of ocular surface disease, or those that cannot be controlled with safe dosing of topical anti-inflammatories, may require reduced dosing or cessation of monoclonal antibody therapy [26, 37]. 

Both dupilumab and tralokinumab are effective and increasingly popular in the management of moderate-to-severe AD. While the literature shows high rates of conjunctivitis and reports of more severe ocular surface disease occurring in association with dupilumab, this has not previously been described with tralokinumab. However, while the underlying pathogenesis for such adverse ocular events is not clear and likely involves both drug-specific effects as well as a predisposition related to AD and/or pre-existing ocular disorders, the most likely underlying mechanisms for drug-induced ocular inflammation are secondary to IL-13 inhibition, which is common to both medications. This case documents the first known report of ulcerative keratitis in association with tralokinumab in a patient with no rheumatologic history and no previously-known ocular surface disease. It highlights the need for both ophthalmologists and prescribing dermatologists/allergists to counsel patients carefully with respect to these medications, to treat early IL-13 inhibitor-associated ocular surface inflammation aggressively, and to be wary of potential progressive keratitis in patients receiving these therapies.

## Data Availability

All data generated or analyzed during this study are included in the published article. Any additional queries may be directed to the corresponding author.

## Abbreviations

AD: Atopic dermatitis
PUK: Peripheral ulcerative keratitis
IL: Interleukin
Ab: Antibody
BCVA: Best-corrected visual acuity
